# An explanatory model for the concept of mental health in Iranian youth

**DOI:** 10.12688/f1000research.12893.2

**Published:** 2018-02-07

**Authors:** Ahdieh Chinekesh, Seyed Ali Hosseini, Farahnaz Mohammadi, Mohammad Esmael Motlagh, Monir Baradaran Eftekhari, Shirin Djalalinia, Gelayol Ardalan

**Affiliations:** 1 Department of Research and Technology, Ministry of Health and Medical Education, Tehran, Iran; 2 Social Determinant of Health Research Center, University of Social Welfare & Rehabilitation Sciences, Tehran, Iran; 3 Department of Adolescents, Youth, and School Health, Bureau of Population, Family, and School Health, Ministry of Health and Medical Education, Tehran, Iran; 4 Department of Pediatrics, Ahvaz Jundishapur University of Medical Sciences, Ahvaz, Iran; 5 Non communicable Diseases Research Center, EMRI, Tehran University of Medical Sciences, Tehran, Iran

**Keywords:** Mental health, youth, Explanatory model, Qualitative method, Iran

## Abstract

**Background: **Mental health is considered as an integral and essential component of overall health. Its determinants and related factors are one of the most important research priorities, especially in adolescents and young people. Using a qualitative approach, the present study aimed to identify factors affecting the mental health of youth in Iran.

**Methods:** In 2017, following content analysis principles, and using semi-structured in-depth interviews, we conducted a qualitative study exploring the opinions of young people about mental health. A targeted sampling method was used, and participants were young volunteers aged 18 to 30 who were selected from Tehran province, Iran. Inclusion criteria for participants was willingness to participate in the study, and ability to express their experiences. Data collection was done with individual in-depth interviews. According to the explanatory model, the interviews were directed toward the concept of mental health and path of causality and auxiliary behaviors.

**Results: **21 young adults participated, who met the study inclusion criteria, of whom 12 participants were male. Their mean age was 24.4 ± 0.41 years and their education varied from primary school to Master’s degree. Mental health was considered as mental well-being and a sense of satisfaction and efficacy, not only the presence of a disease or mental disorder. Based on the opinions of the interviewees, three factors of personal characteristics, family and society are involved in mental health. Individual factors were associated with behavioral and physical problems. One of the most important issues was revealed as tensions in societal and family conflicts. Economic problems and unemployment of young people were also extracted from the social factor.

**Conclusion: **In Iran, social factors such as jobs for the unemployed and job security are considered as important determinants in the mental health of young people.

## Introduction

Mental health is a primary and principal component of health. Based on the definition of the World Health Organization, more than a lack of clinical mental illness, mental health is a sense of happiness that allows individuals to recognize their abilities, adapt to the natural tensions of life, and be efficient and productive resources for their communities ^1^.

Mental health and well-being are essential for personal and social abilities as human beings to think, feel excitement, interact with each other, earn money, and enjoy life. Accordingly, promoting and maintaining mental health can be considered an important and vital concern for individuals, societies, and communities around the world ^2^.

Many biological, social, and psychological factors influence the level and quality of health ^2^. Based on recent evidence, more than 450 million people are suffering from some kind of mental disorder, and there are many psychological problems, around the world ^3^.

Many studies have focused on mental health promotion. Jorum and Wright, in their study in Melbourne, showed that young people believe that interventions for preventions and control of mental disorders could be effective ^4^. There was broad agreement from young people and their parents about what interventions are likely to be helpful and these views applied across the range of disorders presented. These interventions could be described as general and informal sources of help, rather than as specialist mental health services. The most negative views were about psychiatric medications and admission to hospital.

 In 2007, Barry and Jenkins discussed the impacts of many community participation factors in promoting the mental health ^5^. In 2012, Francesco and colleagues examined the role of supporting colleagues in the Clubhouse model using a qualitative method. Through this study, four peer support levels emerged and stressed that peer support would improve ^6^.

In 2004, in Iran, Norbala and colleagues, using the General Health Questionnaire, showed that 21% of the total population suffered from mental illness (25.9% of women and 14.6% of men) ^7^. A related study in 2006 reported that about 2.2% of deaths in women and 1.2% of deaths in men could be attributed to mental disorders ^8^. In 2009, Ebadifard and colleagues conducted a semi-experimental study. The results of this study showed that educational intervention was influenced by attitudes and healthy behaviors in adolescent girls through belief, attitude, and mental and constructive factors ^9^.

Considering the high prevalence of mental health disorders and also their importance and role in the development of related disease ^10^, it is necessary to design effective interventions to improve mental health. Many studies confirmed that improving mental health has considerable association with reducing community social harm ^11, 12^.

In the present study, we were interested in the mental health status of Iranian youth. To investigate this, we used the framework of the explanatory model ^13^, which entails taking notes on a process, according to the concept of mental health, including causes and help-seeking behaviors. This model is mainly used to describe, organize, and manage determinants of welfare disorders ^14^.

## Methods

For this study, to understand the world from youth’s perspectives and their experiences, and to uncover their lived worlds, we conducted individual interviews with young people ^15, 16^. We collected data from September 2016 to May 2017.

### Study participants

Face-to-face in-depth interviews are an easy, flexible method for deeply understanding issues and are mostly used to collect data for explanatory studies ^17^. In a qualitative study, the sample size is based on how many participants it takes to adequately answer the research question. With that aim, and considering the framework of the research question and purpose, the study time frame, and the available resources, we continued purposeful convenience sampling until the point of data saturation ^18^ and no new categories, themes, or descriptions appeared.

Based on the inclusion criteria, we selected participants of different ages from 18 to 30 years old, from different regions of the city, and with different socioeconomic and cultural statuses, using the register list of students in Tehran city universities. We invited participants through their recorded email addresses. This selection was the method of randomization. If participants had not Iranian nationality or if they were beyond the determined age range, they were excluded.

Inclusion criteria for the participants were (a) willingness to participate in the study and (b) ability to describe their experiences.

### Interview guide

The interview guide questions were designed by the scientific committee in accordance with the relevant literature and the study objectives. Toward our aim, we searched for related documents and focused on tools and measurement methods from different approaches. In these regards we searched the main international data bases, Scopus, PubMed and ISI/WOS. These databases cover most multidisciplinary related fields of research and we aimed to access similar studies and research tools. We searched for qualitative studies, research measurements, and guide questionnaires and qualitative questionnaires. The reliability and validity of guide questionnaires are been confirmed by the trust and accuracy that mainly is considered in qualitative research ^19^.

We conducted a pilot study of semi-structured interviews with five young adults, including introducing the entry criteria, and based on the pilot study, we further developed the interview questions. These participants were selected from the universities of Tehran city. We used the list of the university register and then emailed to ask to participate. Young people focused more on the help seeking of mental health problems. After this pilot study we revised some components of interview guide, mostly for more clearance and consistency.

 The main interview guide consisted of six semi-open questions that guided the interview process and data collection ( Supplementary File 1).

### Interviews

We collected the data for this study through deep, semi-structured, face-to-face interviews. At the beginning of the interviews, we explained the research goals and method to the participants. From November 2016 up to February 2017, interviews were planned and conducted by trained experts. Each session lasted for 45–100 minutes (mean duration: 65 minutes). All locations of interviews were selected before the session. The participants were invited through the main researcher. All interviews were conducted in peaceful environments such as special rooms of public libraries or parks with prior agreement of the participants.

Interviews began through general and open-ended questions developed based on the goals of the study, for example, “What are your mental health needs?”; “Can you tell about your experiences of mental well-being?” or “What obstacles have stopped you from having mental health?” The interviews then continued with exploratory questions to clarify the concept and obtain more in-depth information. All conversations were audio recorded with the participants’ permission.

### Data analysis

We performed content analysis of the data collected from the in-depth interviews, ^20^ all of which had been transcribed. To facilitate data analysis, we used Dedoose (version 7.6.6; categorization, constant comparisons, and quotation retrieval). This web-based application, developed at UCLA, is mostly used for analyzing data in qualitative research ^21^.

We read the transcripts to extract the “meaning units.” The coding scheme was theoretically consistent with the context of the study framework, with regard to the concept of mental health including causes and help-seeking behaviors. We extracted the themes and subthemes from the transcripts to generate new codes or modify codes by induction, and we categorized the inductive codes into meaningful topics ^22^.

### Trustworthiness

Trustworthiness in qualitative research has been divided into credibility, dependability, conformability, and transferability ^23– 30^. In this study, we established credibility through the main researcher’s (AC, SAH) prolonged engagement with the subject matter and triangulation of data sources (interviews with youths).

We performed member checking by presenting each interviewee with a summary of the interview to check for accuracy. To address the issues of dependability and conformability throughout the entire research process, the main and local supervisors (Ahdieh Chinekesh, Seyed Ali Hosseini, Shirin Djalalinia) reviewed an audit trail of translated transcripts, related documents for data analysis, and member opinions. For transferability, a complete set of data analysis documents is available on request. This access to the research trail allows other researchers to share the results of this research with others or to repeat the research as closely as possible ^28– 30^.

### Ethical considerations

The study was approved by the University of Social Welfare and Rehabilitation Sciences Ethics Committee in Tehran, Iran (IR.USWR.REC.1395.378). Participation in the study was voluntary, participants were ensured that they could withdraw from the study whenever they wanted, and we obtained written informed consent from all participants. At the beginning of the interviews, we explained the study research goals and methods to the participants and assured them of the confidentiality of the information. We respected the study ethical considerations, including data collection (recording and duplicating interviews), data analysis, and dissemination of results. We collected all information anonymously and used the results only for research purposes.

## Results

The participants consisted of 21 young adults who met the study inclusion criteria; among these, 12 participants were male. The participants’ mean age was 24.4 ± 0.41 years, and their education varied from primary school to master’s degrees. All participants resided in Tehran, although from different geographic regions of Tehran. Table 1 shows the participants’ demographic characteristics.

**Table 1.** Demographic characteristics of participants.

Characteristics	N (%)
**Sex** Female Male	9 (43) 12 (57)
**Grade** Diploma Bachelor Master degree	7 (33) 8 (38) 6 (29)
**Occupation** Unemployed Worker * Employee * Student	3 (14) 4 (19) 6 (29) 8 (38)
Total	21 (100)

* Employee: A person who is employed in the public sector; Worker: a person who works for a private sector and operates under an annual contract.

The results were analyzed under the following themes and subthemes:

### Definition of mental health

The majority of participants believed that mental health is an inner emotional feeling of happiness. They stated that inner peace and satisfaction are among the most important signs of mental health. According to the participants, people who have high self-esteem and who are determined, tolerant, and realistic are satisfied.

Based on the experiences of young people, good mental health cannot be recognized solely by the absence of illnesses and mental disorders such as depression or anxiety. Mental health is also the ability to adapt to one’s environment and surroundings. The participants in this study stated that mental health entailed having healthy individual and social functioning and that it includes skills such as coping, problem solving, communication skills, and stress management.

### Causal pathway of mental health problems

We classified the subthemes of the causes of mental health problems as social, family or personal.

*A. Social factors*

Most participants said unemployment or low-income employment was a major contributor to mental health problems. Unemployment, especially in men, lead to financial problems. On the other hand job insecurity is one of the main sources of stress. People who are unemployed or have low-income jobs are less likely to have mental well-being than do people who are gainfully employed, and they show higher rates of violence, anxiety, aggression, and depression ^31^.

Young people included in this study were pointed out as having a bright future and reported marital problems and the tendency towards high-risk behaviors as important outcomes of unemployment.

Young people in this study highlighted that unemployment, lack of income and lack of entrepreneurship among young people lead to frustration and disappointment. The majority of participants noted the great attention by the authorities to creating jobs.

*‘If you are unemployed, you need to worry about your future and your living costs. Planning for the future is impossible. You’ll be disappointed after a while.’* (Boy, 26, single, bachelor’s degree)

*‘I am looking for a job so I can buy a house and a car.*’ (Boy, 23, single, diploma)

*‘Most young people have no hope of finding right job.*’ (Girl, 24, single, bachelor’s degree)

Young people in this study noted that having a job would prevent high-risk behaviors:

*“The constant presence of young people in the park is due to their unemployment, lead them to cigarettes and other tobacco products”.* (Boy, 27, single, master’s degree)

Young people pointed to having enough income:

“ *Having a job with enough income is one of the most important priorities of mental well-being*.” (Girl, 26, married, bachelor’s degree)

“ *Our problem is that we do not have a good salary system that has enough incentive work. When I started working for the first time, my salary was very low. I was disappointed, and I realized that I had to work two shifts*”. (Boy, 30, married, master’s degree)

Some young people pointed to gender discrimination in finding work:

“ *Girls have less chance than boys for getting a job*”. (Girl, 24, single, bachelor’s degree)

Some young people expressed lack of confidence in the future as one of the most important factors in the lack of mental relaxation:

“ *Society cannot provide this peace of mind for young people who, after completing their course, will find the right work, and we do not have a vision for the future*”. (Boy, 26, single, master’s degree)

The most important issue raised by the young people was that economic problems lead to marital problems:

*“The lack of economic security in society and inflation have an important impact on the issue of marriage. My marriage has been destroyed by economic problems for five years, and we both are still engaged, we cannot solve our economic problems*”. (Boy, 30, married, bachelor’s degree)

*B. Familial factors*

Some participants believed that lack of understanding and coherence in the family, lack of parental attention to young people’s needs, and tension in families such as parental violence can cause emotional difficulties and behavioral problems in children. The participants described the existence of disputes and differences between parents as influences on social security and the need for mental health services. The participants also stated that healthier, more secure family environments have positive effects on family members’ morale and that these favorable effects transfer to society.

*‘Controversy between parents makes me feel dizzy. When I go out of the house after my parent’s dispute, I feel like strangers. I feel like I’m separated. It’s very sad.’* (Boy, 22, single, bachelor’s degree)

*‘Our parents are our supporters, and when they fight and threaten each other, I get tired of life.’* (Girl, 19, single, diploma)

*‘I’m always worried, sometimes my mother speaks and suddenly everything goes wrong and there are arguments and arguments… I leave the house.’* (Boy, 20, single, university student)

The young people referred to factors such as high expectations and disobedience as causes of differences in families:

*‘Sometimes the expectations are too much. The main problem is that everyone has expectations from others, but they do not care what others expect from them. People think they have done well but have not seen anyone good.’* (Boy, 24, single, diploma)

Relationships between family members also affect mental health. Study participants reported the existence of autarchy in the family in the form of patriarchy or matriarchy or collaborative decision-making and respect for the opinions of all family members as the two main types of families:

*‘I learned from childhood to consult, because all decisions in our family are made in consultation with family members.*’ (Boy, 21, single, university student)

*‘In my family, my dad made all the decisions alone and did not consult with family members. Now I am married, but I have no ability to make decisions and I feel that I should obey my husband’s decisions.’* (Girl, 26, married, diploma)

A large number of participants stated that the choice of spouse and the age of marriage played a role in promoting mental health:

*‘As you grow older, making a decision on marriage is harder, and choosing is difficult.*’ (Girl, 25, single, master’s degree)

*C. Individual factors*

According to some participants, behavioral characteristics such as aggressiveness, lying, selfishness, jealousy, pride, irresponsibility are important in mental health problems. From the young people’s perspectives in this study, mentally relaxing requires “managing anger and anxiety” and “gaining happiness” and “hoping for a bright future.”

Most participants stated that forgiveness, patience, anger control, and positive thinking are important for the mental health of young people. Acquiring emotion management skills, especially controlling anger and anxiety, was one of the most common and important mental health needs the young people discussed:

*‘We must not be angry in the tensions of life… learn to be calm.* '(Girl, 22, single, university student)

‘ *The anger in our society is too much. People argue with each other in accidents.’* (Boy, 24, single, diploma)

They also said that exercise is beneficial for mental health:

*‘When exercising, the person feels less jealous and humiliated. As a result, he has better mental health.’* (Girl, 21, single, university student)

### Help seeking

Help seeking can refer to seeking counseling, having social support, or “strengthening the faith in God.” Participants stated that social support is important in personality development and mental health promotion, and they reported receiving social support from various sources such as parents and family members, peers, spouses, and community members. Participants suggested that engaging with friends and peers in activities such as entertainment, hobbies, traveling, and social gatherings were all effective strategies for changing the rhythm of life and facing depression:

*‘If I have a problem I will share with my mother. She understands me and is my best friend.’* (Girl, 21, single, university student)

*‘When I’m sad, I want to be with my friends. They help me and try to understand me.’* (Boy, 24, single, university student)

Most participants referred to the role of religion in achieving peace of mind. These young people reported that they believed in God as a “safe beach” that could be trusted:

*‘When I’m sad, I want help from God. I believe in the existence of a supernatural power, and he helps me.’* (Girl, 21, single, university student)

Most of the young participants in this study recommended learning life skills and communication skills, stating that skills such as stress management, problem solving, and decision-making are effective ways to promote mental health. Participants also recommended counseling during psychological crises, as well as herbal medicines, exercise, and travel as coping strategies:

*‘I think we need to learn how to manage our daily stress, learn the ability to solve a problem, learn communication skills, and learn how to say no. These should be in the form of courses at the school or university for young people.’* (Girl, 24, single, bachelor’s degree)

Although most participants believed in counseling and its benefits, they did note that the cost was a primary barrier to seeking counseling services:

*‘I got help from a psychologist, but because of the high cost, I could not continue.’* (Boy, 21, single, University student)

## Discussion

This was a qualitative study on the perceptions of young people in Tehran about mental health; most participants in this study equated mental health with mental well-being. Based on the results, mental health problems are caused primarily by social, individual, and family factors. Based on the participants’ comments, by controlling daily stress with the help of family and friends, you can manage mental problems at an early stage, but in acute cases, it is necessary to get help from psychologists. The participants also referred to the important role of religion in mental well-being.

The definition of mental health highlights mental well-being. Individuals with good mental health can recognize their abilities, cope with the everyday stress and challenges in life and participate effectively in society ^32^. Health is a multidimensional concept that, in addition to lack of illness and disability, also involves a sense of happiness and well-being ^33^, and the results of this study indicate that many young people are trying to explain mental health positively. They referred to feelings of happiness, hope for the future, and spirituality, which are among the most important indicators of positive mental health ^34^. In various studies, there is a significant relationship between mental health and positive psychological factors such as happiness, hope, and spirituality ^35– 40^.

Our study also showed that most youth attributed mental health to external factors in their social worlds, which was consistent with other studies of participants in Australia, Japan, and Kenya who believed in social causes of poor mental health such as difficult life events and circumstances, financial problems, and traumatic events ^41– 43^. Mental health problems can also be triggered by social circumstances ^44^.

In our results, the young participants perceived psychological symptoms as transient reactions to external stressors. Therefore, young people should receive appropriate training on symptoms of mental illness to be able to recognize mental disorders according to symptoms. In addition, differing approaches to treatment could help professionals to choose appropriate and effective therapies for young people.

We also found that economic problems were considered one of the main causes of poor mental health among the young people in our study. Unemployment and lack of job security cause young people stress and increased tensions related to work, which negatively affect the mental health of young people ^45^. Based on the project COS-RGO, in the field of mental health and economic development, economic changes can increase mental health problems ^46^. Moreover, the results of a national project in Iran showed that economic growth and income inequality among economic variables had the most impact on mental health ^47^.

Among families, the relationship between parents, tensions in the family environment, and divorce affect the mental health of family members ^48^. A multi-method study in the UK showed that parental divorce had a negative effect on adult mental health ^48^. Individual negative traits such as jealousy have been linked with low self-esteem. These behaviors refer to negative thoughts and sense of insecurity, worry, and anxiety ^49^.

The results of this study indicate that help seeking begins with limiting daily stress and that resources such as social support from friends and family and counseling facilitate this process. In this context, religious beliefs are shown to have an important role in achieving peace of mind; reinforcing faith in God has been highlighted as one of the most important sources of peace. Levin *et al*. conducted a study of African Americans in 2005 and found that religion affected physical and mental health ^50^.

As its main strength, we used for this study a well-developed methodology through which we extracted saturated data from in-depth interviews and analyzed the data precisely based on the defined protocol. During the study, we encountered some restrictions, such as the lack of cooperation of some of the invited participants, the diversity of participants' perceptions, and the limitations in generalizing the results.

Given the above, we suggest designing and evaluating participatory interventions for mental health promotion for future studies. Given the strategy outlined, further research on the determinants of mental health in young people in different populations is recommended.

## Conclusion

In Iran, social factors such as jobs for the unemployed and job security for workers have essential roles in youth mental health. Based on the findings of this study, to promote mental health in young people, we propose participatory strategies. In this regard, the youth as a target group will engage with policy makers and researchers to design, develop, and implement mental health programs.

## Data availability

The data referenced by this article are under copyright with the following copyright statement: Copyright: © 2018 Chinekesh A et al.

Data associated with the article are available under the terms of the Creative Commons Zero "No rights reserved" data waiver (CC0 1.0 Public domain dedication). 

Transcripts of the 21 interviews performed with young people (in Persian) are available on request from the corresponding author ( alihosse@gmail.com).

## Supplementary material

Supplementary File 1: Interview guide.
