# Supplementary material for: An explanatory model for the concept of mental health in Iranian youth
Source: F1000Res. 2018 Feb 7;7:52. Originally published 2018 Jan 15. [Version 2] doi: 10.12688/f1000research.12893.2 (PMC5832920; doi:10.12688/f1000research.12893.2)
Supplement: Supplementary file 1 [file f1000research-7-15125-s0000.tgz › 5b244595-e879-457b-bea0-c7f898212713.docx]

| **Interview guide form** | | | |
| --- | --- | --- | --- |
| NO. | The main questions | Probing sub questions | Record events and scenarios |
| 1 | Do you consider yourself a healthy person? (Are you a healthy person?) |  |  |
| 2 | Why do you think you're a healthy person?  Or, Why do not you think you are healthy? | Let's take a discussion of what it needs and the potential?  **Probe 1**: You said that....................  **Probe 2**: Explain what you need to stay healthy? |  |
| 3 | Try to differentiate the dimensions of health in the participants' conversations and treat all aspects of health.  Participant defines every aspect of health. | Probe to explain all aspects of health. And the definition of each dimension.  For example: Do you know about mental health? What are your mental health needs? (A part that the contributor does not mention). |  |
|  | 3.1 Which needs are most important in order? Why?( Arrange the needs in terms of importance.) |  |  |
| 4 | 4.1 What do you do to meet your physical needs? |  |  |
|  | 4.2 What do you need to meet your physical needs? |  |  |
|  | 4.3 What resources do you want to help with these needs? |  |  |
|  | 4.4 How do you think authorities can help you meet these needs? | Or, What would you do if you were responsible for promoting the health of the youth? |  |
| 5 | 5.1 What do you do to meet your mental needs? |  |  |
|  | 5.2 What do you need to meet your mental needs? |  |  |
|  | 5.3 What resources do you want to help with these needs? |  |  |
|  | 5.4 How do you think authorities can help you meet these needs? | What would you do if you were responsible for promoting the health of the youth? |  |
| 6 | 6.1 What do you do to meet your Social needs? |  |  |
|  | 6.2 What do you need to meet your Social needs? |  |  |
|  | 6.3 What resources do you want to help with these needs? |  |  |
|  | 6.4 How do you think authorities can help you meet these needs? | What would you do if you were responsible for promoting the health of the youth? |  |
| 7 | 7.1 What do you do to meet your Spiritual needs? |  |  |
|  | 7.2 What do you need to meet your Spiritual needs? |  |  |
|  | 7.3 What resources do you want to help with these needs? |  |  |
|  | 7.4 How do you think authorities can help you meet these needs? | What would you do if you were responsible for promoting the health of the youth? |  |
| 8 | What factors help your need to be met? |  |  |
| 9 | What factors prevent your need to be met? |  |  |
| 10 | What is the most important thing to do to meet your health needs? |  |  |
|  | 10.1 Who should help to meet these needs? |  |  |
|  | 10.2 What organizations should work to meet these needs? |  |  |
