# Supplementary material for: An explanatory model for the concept of mental health in Iranian youth
Source: F1000Res. 2018 Feb 7;7:52. Originally published 2018 Jan 15. [Version 2] doi: 10.12688/f1000research.12893.2 (PMC5832920; doi:10.12688/f1000research.12893.2)
Supplement: Peer review history

# Peer review: Referee response for version 2

**DOI:** 10.5256/f1000research.15125.r29803

**Published:** 2018-02-28

**Authors:** Nourossadat Kariman

**Affiliations:** 1 Midwifery and Reproductive Health Research Center, School of Nursing and Midwifery, Shahid Beheshti University of Medical Sciences, Tehran, Iran

Thanks for sharing this manuscript with me. I read it carefully. Based on my previous related experiences, I can confirm that it benefited from well-developed methodology following by well conducted processes. The qualitative statistical analysis and its interpretation is appropriate. The results are presented clearly and accurately. Conclusions are supported by the results. This article contributes significantly to the problems of youth in Iran and has well understood youth's views.

I have read this submission. I believe that I have an appropriate level of expertise to confirm that it is of an acceptable scientific standard.

# Peer review: Referee response for version 2

**DOI:** 10.5256/f1000research.15125.r30655

**Published:** 2018-02-19

**Authors:** Sara Esmaelzadeh Saeieh

**Affiliations:** 1 Social Determinants of Health Research Center, Alborz University of Medical Sciences, Karaj, Iran

I have read this submission. I believe that I have an appropriate level of expertise to confirm that it is of an acceptable scientific standard.

# Peer review: Referee response for version 1

**DOI:** 10.5256/f1000research.13975.r29804

**Published:** 2018-01-23

**Authors:** Sara Esmaelzadeh Saeieh

**Affiliations:** 1 Social Determinants of Health Research Center, Alborz University of Medical Sciences, Karaj, Iran

Please correct grammar in the paper

 Please provide further details about time of sampling, situation and environment of the interview.

 Who is doing the interview?

 Was the interviewer an expert?

 We suggest that results of qualitative study include theme (category) sub them (sub category) and codes of study are mentioned in table.

 Please omit extra explanation about methods and only mention the methods of your study.

 Please explain about the type of content analysis.

I have read this submission. I believe that I have an appropriate level of expertise to confirm that it is of an acceptable scientific standard, however I have significant reservations, as outlined above.

# Response

**Published:** 2018-01-27

**Authors:** ahdieh chinekesh

**Affiliations:** Social Determinants of Health Research Center, University of Social Welfare and Rehabilitation Science, Iran

Dear chief editor of F1000research

Thanks for your comments. All of them have been considered in revised upload formatted carefully. 

Ahdieh Chinekesh

# Response

**Published:** 2018-02-04

**Authors:** ahdieh chinekesh

**Affiliations:** Social Determinants of Health Research Center, University of Social Welfare and Rehabilitation Science, Iran

In the revised version of this article, we have included two sentences in the first paragraph of the ‘Interviews’ section, providing more detail on how they were conducted.
